# Modern Mental Disorders in a Medico-Legal Point of View

**Published:** 1859-11

**Authors:** 


**Modern Mental Disorders in a Medico-Legal point of view.**

In our department of selections may be found a brief review of several contributions recently made in England, to our knowledge, in reference to the best mode for the prevention and treatment of mental disorders, in which may be found some valuable suggestions.

From this "Review," we learn that Dr. Robinson, (the author of one of the works referred to) differs in one important point from some other recent writers upon Insanity, who have maintained the reality of a morbid condition, to which the designation "Moral Insanity," has been given, and which is said to be characterized by a morbid perversion of the feelings and active powers, without any illusion or erroneous conviction impressed upon the understanding, and has been regarded as not incompatible with an apparently unimpaired state of the intellectual faculties. An example of such a peculiar form of insanity being furnished by an individual (such as Huntington) who manifested a controlling impulse to the indulgence in libertinism, fast horses, costly wines, cards, billiards, and other fashionable luxuries, or vices at the expense of a bank or individual with whose funds he had been entrusted, exhibiting all the while, and for years before *detection*, not only no evidence of "Mental Insanity," but a remarkable degree of astuteness in concealing his *villainy*. We fully agree with Dr. Robinson in the idea, that while the moral sense of the individual who retains his intellectual faculties may be impaired, yet there are few under such circumstances in whom the powers of the will, the reason and the conscience, are so far undermined as to render them altogether incapable of self-control, and consequently irresponsible. And while his suggestion of the substitution of a *reformatory* for a *retaliatory* principle as the ground of the punishment inflicted upon such persons by the *law*, is a good one, we cannot too carefully guard the distinction between the *natural* tendencies of men to vice and crime, and that moral infirmity which may be attributed to a *morbid* condition, which in the former case deserves the infliction of punishment, and in the latter only requires *restraint* upon grounds of social security, and the future welfare of the individual. That there are large numbers of individuals of unaffected intellect who have so far lost the control of the will, that in a certain sense they may be regarded as incapable of resisting criminal impulses or vicious cravings, (as in the case of the habitual drunkard) is doubtless true, but if such persons are to be regarded as irresponsible, and their moral weakness is to be admitted as exculpatory so far as the retaliatory principle of the law is concerned, which would seem to be reasonable in the case of confirmed inebriates, at least, the law having legalized the means and given all the assistance in its power to make them so—they should yet, most unquestionably, for the safety of society and their own welfare, become the subjects of a humane but decided and reformatory discipline and restraint in an Asylum; and in the case of the unfortunute individual laboring under the *uncontrollable* thirst for liquor, should be supported by the same government that had legalized a traffic, the constant result of which is, the degradation and ruin of the bodies and minds of our race. Whenever the inconsistency which the law now presents, (licensing the manufactories of drunkards, and yet punishing the poor victim who commits crime under the influence of intoxication) is removed, then there will be unquestionably propriety in adding drunkenness to the list of crimes and an aggravation of any offence committed in that condition: until that period, there would seem, in a medico-legal point of view, at least, great fitness in considering, not only the condition of the mind brought about by the long continued and excessive use of alcohol, as an irresponsible state, but the *thirst for liquor* and the *immediate effect* of indulgence in it (*drukenness*) as only other forms of mental disorder affording equal immunity from retaliatory punishment for crime committed under such circumstances.

In conclusion, we would remark that our attention has been directed to this subject recently, in connection with a case of melancholy interest, occurring in the Superior Court of Fulton county, Ga., in which the plea of insanity was unsuccessfully made the defence against the charge of murder, the convicted individual having been proved to be habitually intemperate and in that condition unusually violent and uncontrollable, which was attributed by the defence to injuries received on the head a number of years before the unfortunate occurrence. He was convicted upon the ground that he was regarded as a sane man, and one of more than ordinary intelligence when free from intoxicating liquors, and the crime having been committed when he was proved to have been in a state of drunkenness, the law regarded as not only no excuse for but an aggravation of the crime, that condition having been voluntarily imposed upon himself, with a full knowledge of its effects.

While, therefore, the decision in this case, was in strict accordance with the *law*, and as a naked question of the responsibility of individuals for acts committed in a condition (though irrational) which has been voluntarily brought upon themselves, should in our judgment, commend itself to the sense of right and justice of every good citizen, yet, practically, the question arises: Has the law any moral right to visit *retaliatory* punishment upon men for the indulgence in, and the consequences of, that which it legalizes, and which is one of the regular sources of its revenue? It seems to us that it would be far more in accordance with an enlightened justice and philanthropy for the law to consign such an individual to the *reformatory* punishment and discipline of a forcible confinement in an "Asylum of Drunkards," supported at the expense of the government, which, under our present laws, is largely responsible for the drunkenness and its consequent insanity and crime, which curses the land.

